# Investigation of piwi-interacting RNA pathway genes role in idiopathic non-obstructive azoospermia

**DOI:** 10.1038/s41598-017-17518-4

**Published:** 2018-01-09

**Authors:** Zeeba Kamaliyan, Sara Pouriamanesh, Mohsen Soosanabadi, Milad Gholami, Reza Mirfakhraie

**Affiliations:** 1 Department of Medical Genetics, School of Medicine, Shahid Beheshti University of Medical Sciences, Tehran, Iran; 2 Faculty of Medicine, Semnan University of Medical Sciences, Tehran, Iran; 3 Genomic Research Center, Shahid Beheshti University of Medical Sciences, Tehran, Iran

## Abstract

Genes involved in piwi-interacting RNAs (piRNAs) pathway have an essential role in spermatogenesis. HIWI and TDRD proteins are critical for piRNA biogenesis and function. Therefore, Mutations and polymorphisms in *HIWI* and *TDRD* genes may play role in male infertility. The aim of the present study was to investigate the role of *HIWI2* rs508485 (T>C) and *HIWI3* rs11703684 (C>T) polymorphisms and mutational analysis of TDRD5 gene in idiopathic non-obstructive azoospermia in a case-control study including 226 non-obstructive azoospermia patients and 200 fertile males. Genotyping for both polymorphisms was performed using Tetra-Primer ARMS PCR. Mutation analysis of *TDRD5* gene was done using multi-temperature single strand conformation polymorphism technique (MSSCP). The frequency of rs508485TC genotype was significantly different in the studied groups (P = 0.0032; OR = 2.12; 95% CI, 1.29–3.48). In addition, the genotype frequencies showed a significant difference under dominant model (*P* = 0.005; OR = 2.79; 95% CI, 1.22–3.13). No mutation was detected in the Tudor domain of the *TDRD5* in the studied patients. In conclusion, we provide evidence for association between genetic variation in the *HIWI2* gene and idiopathic non-obstructive azoospermia in Iranian patients. Therefore, piRNA pathway genes variants can be considered as risk factors for male infertility.

## Introduction

Infertility is defined as the failure to make a clinical pregnancy after 12 months or more of regular unprotected sexual intercourse. According to the worldwide statistics, infertility affects 10–15% of couples and almost in the half of the cases, men are responsible^1^. Azoospermia is the most common reason for male infertility with a prevalence of 10–15% in infertile men and 1% of all men^2^. Although several diverse reasons are mentioned for azoospermia, the main reason is still unknown. The most of our knowledge about the possible link between a gene and male infertility is the result of the gene knockout studies in animal models and the only way to confirm this relationship is to study the candidate genes structure and function in human diseases^1^.

Recent studies have revealed piRNA pathway as a new essential pathway for spermatogenesis. The genes involved in this pathway are expressed abundantly and solely in germline cells. This class of non-coding RNAs forms a retrotransposon silencing complex in germline via binding to a different subtype of Argonaut proteins^3–5^. Many studies showed that piRNAs are crucial for the differentiation and specificity of male germ line. In addition to cutting and degradation, they can repress transposons by histone modifications and DNA methylation^6,7^. Although piRNAs are expressed both in testis and ovaries, only mutant male mice for these genes become sterile probably due to the overexpression of transposons in the germline^8,9^.

The pathway function depends on P-element Induced Wimpy testis proteins (PIWIs) and Tudor domain-containing proteins (TDRDs). PIWIs are the most important proteins in this pathway that play an important role in piRNA biogenesis and function. In humans, this subclass of Argonaut protein family includes *HIWI*, *HIWI2*, *HIWI3* and *HILI*. Knockout studies in mice have revealed that silencing of these genes results in meiotic arrest and male sterility^10–13^. In addition, it has been suggested that single nucleotide polymorphisms (SNPs) including rs508485 and rs11703684 in *HIWI* genes are associated with the risk of male infertility in different ethnic groups^14,15^.

TDRD proteins act as mediators or adaptors for protein-protein interactions in the piRNA pathway via binding to the dimethylated arginines of PIWI proteins by their Tudor domains^16^. TDRDs consist of 12 members in humans and animal model studies have proved that mutation in any of these genes blocks the spermatogenesis^16–18^. Previous studies on the exact role of *TDRD5* in transposon repression, chromatid body assembly and spermiogenesis in mice proved that the gene expression is essential for male fertility^19,20^.

Considering the functional and physiological importance of *HIWI* and *TDRD* genes in male fertility and the results of our previous pilot study concerning the association between rs508485 and azoospermia^21^, the aim of the present study was to analyze the association between *HIWI2* rs508485 (T>C) and *HIWI3* rs11703684 (C>T) polymorphisms with the risk of azoospermia in a larger sample of Iranian infertile men with idiopathic non-obstructive azoospermia. Moreover, we investigated mutations in the functional Tudor domain of the *TDRD5* in the piRNA pathway in the patients.

## Results

Twenty-six (13%) patients showed Y chromosome microdeletions and were excluded from the study. Outer primers in all PCR reactions for rs508485 amplified a common 338 bp band. The T and C alleles generated 253 bp and 141 bp PCR products, respectively. For rs11703684 besides a common 264 bp PCR product, the T and C alleles amplified 190 bp and 126 bp PCR products, respectively. The genotype distribution of both polymorphisms in the studied groups was all in Hardy-Weinberg equilibrium. The genotype and allele frequencies for *HIWI2* rs508485 and *HIWI3* rs11703684 polymorphisms in the cases and controls are summarized in Table 1. The frequency of rs508485TC genotype was significantly different in the studied groups (*P* = 0.0032; OR = 2.12; 95% CI, 1.29–3.48). In addition, the genotype frequencies showed a significant difference under dominant model (*P* = 0.005; OR = 2.79; 95% CI, 1.22–3.13). There was no significant difference in allele and genotype frequencies between patients and controls for rs11703684 polymorphism. In addition, no mutation was detected in the Tudor domain of *TDRD5* gene.

**Table 1:** Allele and genotype frequencies of HIWI2 rs508485(T>C) and HIWI3 rs11703684(C>T) polymorphisms in non-obstructive azoospermia patients and controls.

Genotype/Allele	Cases n (%)	Controls n (%)	OR (95% CI)	*P-Value*
***HIWI2*** **rs508485(T>C) polymorphism**				
T/T	36 (18)	60 (30)	1 (reference)	
T/C	113 (56.50)	89 (44.50)	2.12 (1.29–3.48)	0.0032
C/C	51 (25.50)	51 (25.50)	1.67 (0.95–2.94)	0.08
C/C+T/C vs. T/T			2.79 (1.22–3.13)	0.005
C/C vs. T/C+T/T			1.00 (0.64–1.58)	0.99
T	185 (46.25)	209 (52.25)	0.79 (0.60–1.04)	0.09
C	215 (53.75)	191 (47.75)	1.27 (0.96–1.68)	0.09
***HIWI3*** **rs11703684(C>T) polymorphism**				
C/C	119 (59.50)	120 (60)	1 (reference)	
C/T	76 (38)	75 (37.50)	1.02 (0.68–1.54)	0.92
T/T	5 (2.50)	5 (2.50)	1.01 (0.28–3.57)	0.99
T/T+C/T vs. C/C			1.02 (0.68–1.52)	0.92
T/T vs. C/T+C/C			1.00 (0.29–3.51)	0.99
C	314 (78.50)	315 (78.75)	0.99 (0.70–1.38)	0.93
T	86 (21.50)	85 (21.25)	1.01 (0.72–1.42)	0.93

## Discussion

It was well known that several protein coding genes are involved in the process of spermatogenesis, however, recent studies using gene knockout animal models and genome wide association studies (GWAS) have revealed that non-coding RNAs are also implicated in this process^22,23^. PiRNAs are among non-coding RNAs that are essential for male germ line development. PIWI and TDRD proteins contribute to the biogenesis and functions of piRNAs and are essential for the progression of spermatogenesis^24^. Hence, mutations and polymorphisms in these coding genes can play a vital role in spermatogenesis defects resulting in male infertility.

In the present study, we have analyzed the association of rs508485 and rs11703684 polymorphisms in *HIWI* genes with the risk of idiopathic non-obstructive azoospermia in Iranian infertile men. The association was observed between rs508485 (T>C) and increased risk of azoospermia in our studied population. Single nucleotide variations could hypothetically influence gene expression and/or protein structure by altering cis-acting elements, RNA transcript stability, or RNA splicing. The rs508485 locates in 3′ UTR of *HIWI2* gene and due to its position it may affect mRNA stability or may alter the binding affinity of regulatory miRNAs.

We used several databases including, mirBase (http://microrna.sanger.ac.uk), miRNASNP (http://www.bioguo.org/miRNASNP/index.php), MicroSNiPer http://vm24141.virt.gwdg.de/services/microsniper/index.php), TargetScan (http:// targetscan.org/vert_71/) in order to predict miRNAs that target *HIWI2* 3′ UTR region. The predicted miRNAs are including hsa-miR-4686, hsa-miR-3686, hsa-miR-4652-3p, and hsa-miR-215-3p. Among these hsa-miR-215 is transcriptionally regulated by p53 and is capable to induce cell cycle arrest^25^. Moreover, hsa-miR-215 is among over 200 miRNAs discovered in the human epididymis that potentially play an important role in apoptosis, stress response, and differentiation of the epididymal epithelium and therefore male infertility^26,27^.

According to HaploReg v4 (http://www.broadinstitute.org/mammals/haploreg/haploreg.php), it is suggested that the mentioned polymorphism may also influence binding affinity of several transcription factors, including CEBPG, Fox, Hoxa9 and GATA and hence affects the gene expression level. Consistent with this hypothesis, Hadziselimovic *et al*., showed that the expression of several transposon silencing genes, including *DDX4, MAEL, MOV10L1, HILI, HIWI2*, and *TDRD9* genes was silenced or reduced in cryptorchid boys with high risk of azoospermia. They concluded that this altered expression might be responsible for the massive germ cell loss in these patients^28^. Moreover, hypermethylation of *HILI* and TDRD1 has been reported in patients with spermatogenic failure^29^.

Rs11703684 is an exonic variant that changes amino acid codon 471 in HIWI3 protein from Val to Ile. This variant may change the binding affinity of the E2F transcription factor. According to the literature, there are only two studies concerning the role of HIWI gene family polymorphisms and susceptibility to male infertility. Gu *et al*. showed that that genetic variations in *PIWI* gene family are more likely to be associated with oligozoospermia, but not azoospermia in Chinese population. They concluded that *PIWI* gene variations might only have a relatively modest effect on spermatogenesis^30^. However, Munoz *et* al. found that rs508485 is associated with severe maturation arrest that leads to azoospermia in Spanish men. Therefore, consistent with the present study, their result supports the role of PIWI proteins in spermatogenesis and self-renewal of germ stem cells^15^. The observed controversy between the previous and present studies is mainly due to different genetic backgrounds and ethnicity in the studied populations.

In this study, no mutations were detected in the Tudor domain of TDRD5 in our patients. TDRDs can be classified in two groups: genes that are expressed in the embryonic stage in the male germline, including *TDRD1* and *TDRD9*, which are necessary for meiosis progression due to their transposon silencing function^31,32^. The second group, including, *TDRD4* and *TDRD6* that is expressed at birth during pachytene and is necessary for spermiogenesis^33,34^. Yabuta and his colleagues proved that *TDRD5* is the only member of this family that functions in both meiotic and post meiotic stages and its loss results in lack of sperm maturation, transposons overexpression and sterility^20^. To the best of our knowledge, this is the first study concerning the investigation of possible mutations in the Tudor domain of *TDRD5* in non-obstructive azoospermia infertile men. Although no mutation was detected in exons 10 and 11 that code the functional Tudor domain, screening other exons of this gene can be considered in future studies.

In conclusion, this study showed that genetic variants in piRNA pathway genes might predispose to spermatogenesis defects. To better understand the relationship between piRNA pathway and male infertility studying other genes of this pathway in different types of male infertility should be considered.

## Materials and Methods

In a case-control study, 426 subjects including 226 non-obstructiveazoospermic patients and 200 proven fertile men were enrolled. All patients had normal karyotype and aged between 21 and 62 (mean ± SD = 34.53 ± 7.65 yrs.). The patients were referred from the Yazd Research & Clinical Center for infertility, Kowsar Infertility Treatment Center and IVF Department of Day hospital. Hormone analysis including follicle-stimulating hormone (FSH) and luteinizing hormone (LH) was also performed. The mean values for FSH and LH concentration in patients were 34.75 mIU/ml and 14.62 mIU/ml, respectively. Two hundred age-matched (mean ± SD = 33.6 ± 8.16 yrs.) fertile men who had at least one child with no history of requiring assisted reproduction technology were considered as control group. Semen analysis was assessed according to the World Health Organization (1999) criteria^35^. Urological examination was performed in the patients for anatomical integrity of genital system. Patients with anatomic disorders of genitalia, testis neoplasms, obstruction, congenital bilateral absence of vas deferens, and chromosomal abnormalities were excluded. Informed written consent was obtained from the participants. The study was carried out in accordance with the approved guidelines of the WMA Declaration of Helsinki. The ethics committee of Shahid Beheshti University of Medical Sciences (SBMU) approved the study protocol (Code: IR.SBMU. MSP.REC.1395.398).

### Detection of Y chromosome microdeletions

Peripheral blood samples were collected from the study participants in EDTA tubes and genomic DNA was extracted by using M&D DNA extraction kit (Shahid Beheshti University of Medical Sciences, Iran). In order to exclude the role of Y chromosome microdeletions, a series of eight sequence tagged site markers (STS) located on Yq11 were selected for the detection of submicroscopic deletions in the AZFa, AZFb and AZFc regions using Multiplex PCR^36^.

### Genotyping of *HWI2* rs508485 (T>C) and *HIWI3* rs11703684(C>T)

We used tetra-primer amplification refractory mutation system-PCR (4P-ARMS-PCR) method^37^, which applies two pairs of primers, to analyze *HWI2* rs508485 (T>C) and *HIWI3* rs11703684(C>T) genotypes. Primers were designed by using Primer1 online software (http://primer1.soton.ac.uk/primer1.html). PCR primers used for rs508485 were including, F-outer508: 5′ AAAAGATTGAGCTTAGTTTTCATGTCTAG 3′, R-outer508: 5′ CACATGATGTTCTGAACTTTATTTTCA 3′, F-inner508 for C allele: 5′ ATAAGTGTTTGCGTGATATTTTGATTAC 3′ and R-inner508 for T allele: 5′ GTGGTGGGAATTAGACTCTGTTTATATA 3′. Each PCR reaction contained 100 ng of DNA, 10 µl Taq DNA Polymerase 2X Master Mix Red (Amplicon, Denmark), 10 pmol/µl of inner primers, and 5 pmol/µl of outer primers in a final volume of 25 µl. Amplification was carried out on a GeneTouch (BIOER, China) with the following program: 95 °C by 5′ for primary denaturation, three steps of 95 °C by 30′′, 50 °C by 45′′ and 72 °C by 45′′ for 32 cycles and final extension of 72 °C by 5′.

PCR primers for rs11703684 were including, F-outer117: 5′ TCCTTTTTGCTTTACTCTTCATTTGACC 3′, R-outer117: 5′ TTGGAATAGAAGGAAATTGCCTTGCA 3′, F-inner117 for T allele: 5′ TGTACGATGTTTGCGTTTTTCAACAT 3′ and R-inner117 for C allele: 5′ CAATTTTTTGTCAGTCCCGGGAATAG 3′. Again, each PCR reaction contained 100 ng of DNA, 10 µl Taq DNA Polymerase 2X Master Mix Red (Amplicon, Denmark), 10 pmol/µl of inner primers, and 5 pmol/µl of outer primers in a final volume of 25 µl. PCR program that was performed for rs11703684 in a GeneTouch (BIOER, China) included: 95 °C by 5′ for primary denaturation, three steps of 95 °C by 30′′, 56 °C by 45′′ and 72 °C by 45′′ for 32 cycles and final extension of 72 °C by 5′. PCR products were subjected to electrophoresis on 2% agarose gel prepared in 0.5X TBE, stained with RedSafe (iNtRON, Korea). For further confirmation,ten percent of the samples were sequenced by using an ABI 3730xl DNA analyser (Macrogen, Korea).

For mutation detection in Tudor domain of *TDRD5* gene, exons 10 and 11 and their adjacent intronic sequences were amplified separately with specific primers: F-Td10: 5′ TTTCTTCCCCTATGGATTTCTCTGT 3′ and R-Td10: 5′ ACCCCACAGAGCTTCAACAC 3′ for exon 10; F-Td11: 5′ TGGGTCATGCTAAGAGGTGC 3′ and R-Td11: 5′ TGACTTCTTTGAAGGCTGGTTCT 3′ for exon 11. Each PCR reaction contained 100 ng of DNA, 10 µl Taq DNA Polymerase 2X Master Mix Red (Amplicon, Denmark), 10 pmol/µl of each primer in the a final volume of 25 µl. Amplification was carried out in a GeneTouch (BIOER, China) with the following program: 95 °C by 5′ for primary denaturation, three steps of 95 °C by 30′′, 53 °C for exon10/ 55 °C for exon11 by 45′′ and 72 °C by 45′′ for 32 cycles and final extension of 72 °C by 5′. Mutation screening was performed using multi-temperature single strand conformation polymorphism (MSSCP) technique on a 10% undenaturing polyacrylamide gel^38^. Samples with altered banding pattern were sequenced on an ABI 3730XL automated DNA sequencer (Macrogen, Seoul, Korea).

### Statistical analysis

Allele and genotype frequencies and the Hardy-Weinberg equilibrium were calculated by chi-squared test test using MedCalc online software available from http://www.medcalc.org/calc/odds_ratio.php. *P*-Value of < 0.05 was considered to be statistically significant.

## Electronic supplementary material

Supplementary data
