# Supplementary material for: Investigation of piwi-interacting RNA pathway genes role in idiopathic non-obstructive azoospermia
Source: Sci Rep. 2018 Jan 9;8:142. doi: 10.1038/s41598-017-17518-4 (PMC5760646; doi:10.1038/s41598-017-17518-4)
Supplement: Supplementary file 1 — Supplementary data [file 41598_2017_17518_MOESM1_ESM.doc]

**Investigation of piwi-interacting RNA pathway genes role in idiopathic non-obstructive azoospermia**

Zeeba Kamaliyan, Sara Pouriamanesh, Mohsen Soosanabadi, Milad Gholami, Reza Mirfakhraie

1 2 3 4 5 6 7 8 9


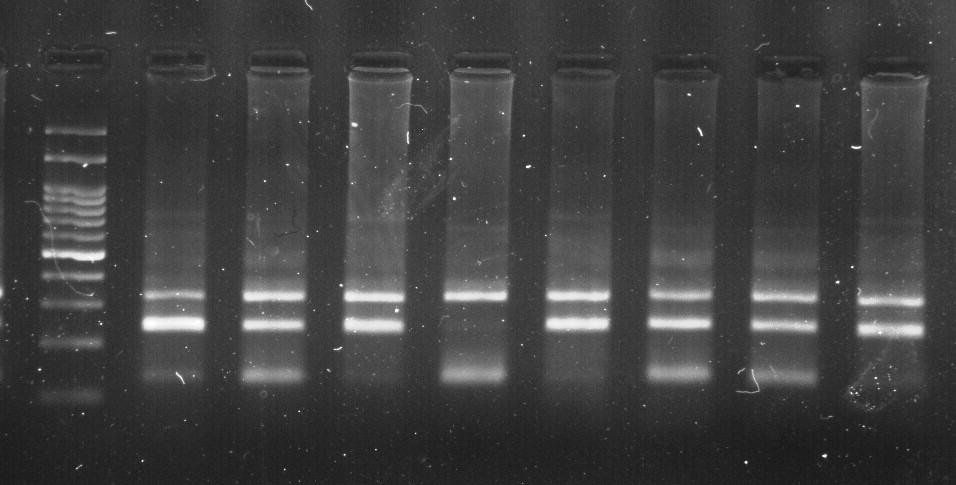


**Figure1:** Agarose gel showing Tetra-ARMS PCR genotyping results for rs508485. Lanes 2, 3, 7 and 8: TC, lanes 4, 6 and 9: TT, lane 5: CC genotypes, lane 1: 100 bp DNA ladder.

**338 bp**

**253 bp**

**141 bp**

1 2 3 4 5


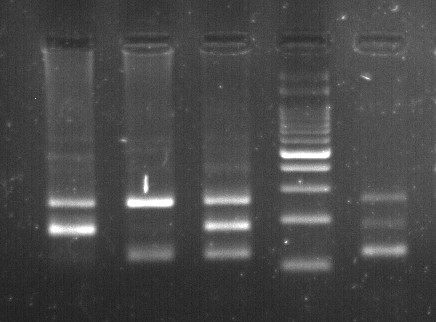


**264 bp**

**190 bp**

**141 bp**

**Figure2:** Agarose gel showing Tetra-ARMS PCR genotyping results for rs11703684. Lane 1: TT, lane 2: CC, lanes 3 and 5: TC genotypes, Lane 4: 100 bp DNA ladder.

**1** **2 3 4 5 6 7**  **8 9 10 11**


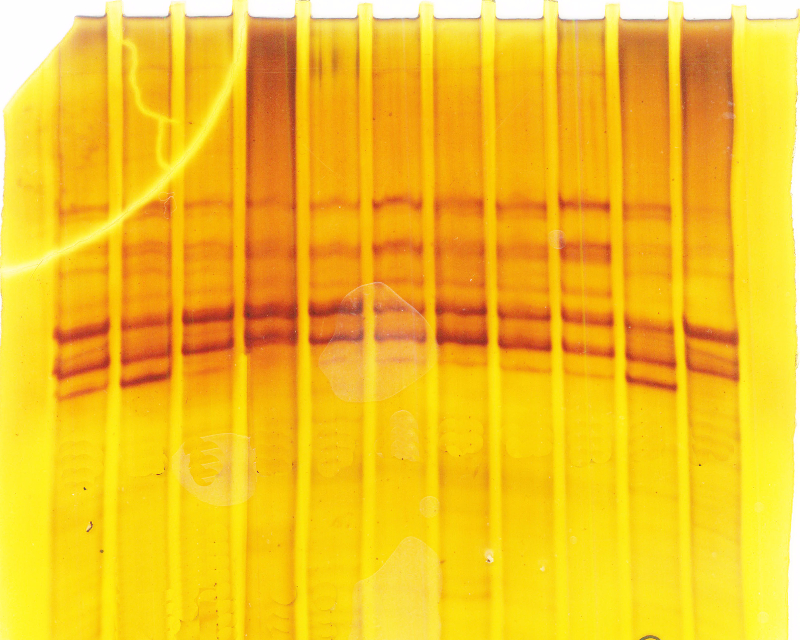


**Figure3:** MSSCP results of TDRD5-exon9 on 10% polyacrylamide gel. Samples 4 and 9 are normal controls, other samples are azoospermic patients.

**1** **2 3 4 5 6 7**  **8 9 10 11**  **12**


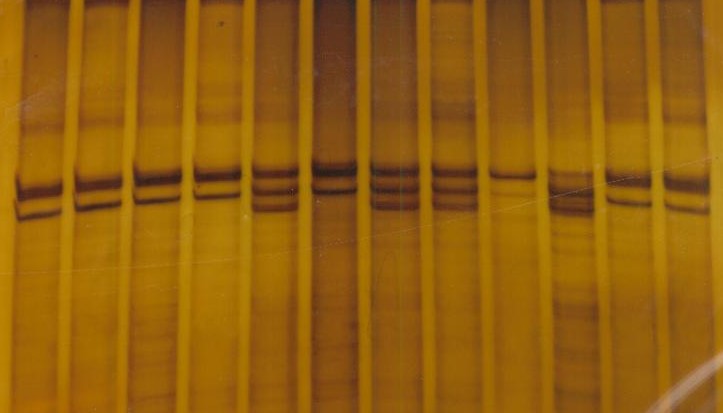


**Figure4:** MSSCP results of TDRD5-exon10 on 10% polyacrylamide gel. Sample 6 is normal control, other samples are azoospermic patients.
